# Understanding FRET as a Research Tool for Cellular Studies

**DOI:** 10.3390/ijms16046718

**Published:** 2015-03-25

**Authors:** Dilip Shrestha, Attila Jenei, Péter Nagy, György Vereb, János Szöllősi

**Affiliations:** 1 Department of Biophysics and Cell Biology, University of Debrecen, Egyetem tér 1, Nagyerdei Krt. 98, Debrecen 4032, Hungary; E-Mails: aagaman@gmail.com (D.S.); jenei@med.unideb.hu (A.J.); nagyp@med.unideb.hu (P.N.); vereb@med.unideb.hu (G.V.); 2 MTA-DE Cell Biology and Signaling Research Group, Faculty of Medicine, University of Debrecen, Egyetem tér 1, Debrecen 4032, Hungary

**Keywords:** FRET, Methods for measuring FRET, Fluorescence intensity, Fluorescence lifetime, Anisotropy, Major Histocompatibility Complex (MHC), CD1d, IL2, IL15, Immune synapse, ErbB

## Abstract

Communication of molecular species through dynamic association and/or dissociation at various cellular sites governs biological functions. Understanding these physiological processes require delineation of molecular events occurring at the level of individual complexes in a living cell. Among the few non-invasive approaches with nanometer resolution are methods based on Förster Resonance Energy Transfer (FRET). FRET is effective at a distance of 1–10 nm which is equivalent to the size of macromolecules, thus providing an unprecedented level of detail on molecular interactions. The emergence of fluorescent proteins and SNAP- and CLIP- tag proteins provided FRET with the capability to monitor changes in a molecular complex in real-time making it possible to establish the functional significance of the studied molecules in a native environment. Now, FRET is widely used in biological sciences, including the field of proteomics, signal transduction, diagnostics and drug development to address questions almost unimaginable with biochemical methods and conventional microscopies. However, the underlying physics of FRET often scares biologists. Therefore, in this review, our goal is to introduce FRET to non-physicists in a lucid manner. We will also discuss our contributions to various FRET methodologies based on microscopy and flow cytometry, while describing its application for determining the molecular heterogeneity of the plasma membrane in various cell types.

## 1. Background

The major constituents of all living beings are nano-sized molecular species like lipids, proteins, carbohydrates and nucleic acids [1]. These molecules are constantly in communication with each other through complex yet specific interactions in a crowded molecular environment regulating the biological functions in an organism [2,3]. Therefore, uncovering the secrets in the way these molecules function requires an approach that would enable monitoring the molecular actions at nanometer scale. One of the first described methods capable of reaching nanometer resolution was based on the principle of Förster Resonance Energy Transfer (FRET), which nowadays is also referred to as fluorescence resonance energy transfer mainly owing to the usage of fluorescence-based probes for FRET applications. In the 1920s, Perrin first introduced the concept of dipole–dipole interaction and distance dependent transfer of energy without molecular collision [4]; however, the accurate quantitative theory of FRET occurring between two closely juxtaposed molecular species was correctly described by Theodor Förster in 1948 [5]. Experimental verification of this theory, primarily the distance dependence of FRET, was proven only in the late 1960s [6,7]. Importantly, with his insightful seminal papers, Lubert Stryer popularized FRET as a “Spectroscopic Ruler” [6,8]. The application of FRET in biology gained momentum only in the last 20 years after huge technical advances in physical and biological sciences, integration of FRET with easy-to-use fluorescence based instruments and simple classification of the various modalities of FRET measurements.

## 2. Introduction

**Figure 1:**
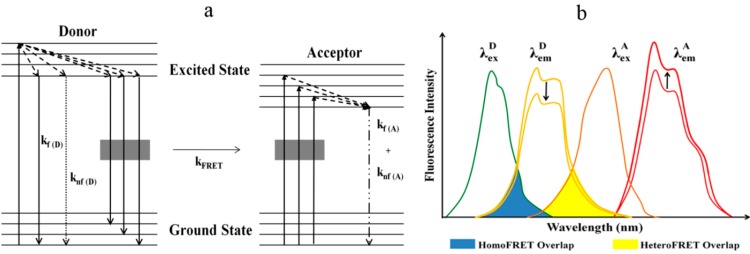
(**a**) The figure shows the Jablonski diagram demonstrating mechanism of Förster Resonance Energy Transfer (FRET). On absorption of energy, electrons in both donor and acceptor are excited from the ground state to an excited state, and they lose energy as fluorescence with rate constant k_f(D)_ for donor or k_f(A)_ for acceptor and non-fluorescence mechanisms with rate constant k_nf(D)_ for donor or k_nf(A)_ for acceptor. On the occurrence of FRET, excited energy of the donor is also lost via FRET to an acceptor with rate constant k_FRET_; and (**b**) Spectral overlap: The absolute requirement of FRET is illustrated in this figure. The symbols “${\text{λ}}_{\text{ex}}^{\text{D}}$” and “${\text{λ}}_{\text{em}}^{\text{D}}$” or “${\text{λ}}_{\text{ex}}^{\text{A}}$” and “${\text{λ}}_{\text{em}}^{\text{A}}$” indicate excitation (λ_ex_) and emission spectra (λ_em_) of donor and acceptor fluorophores respectively with upper index letters denoting fluorophores. Essential spectral overlap in the case of heteroFRET (yellow) and homoFRET (blue) is also highlighted in the figure.

FRET is a collision-free, but distance-dependent photophysical process where radiationless transfer of energy occurs from an excited donor (D) fluorophore to a suitable acceptor (A) protein or fluorophore via long-range dipole–dipole coupling mechanism. Donor absorbs energy at shorter wavelength whereas acceptor has energy absorption at longer wavelength [9,10]. FRET occurs over interatomic distances due to resonance-based interaction of chromophores without transmission of photons from donor to acceptor species. Therefore, it is inaccurate to use fluorescence with the acronym FRET, since fluorescence involves emission of photons. At distances below 1 nm, collision between donor and acceptor would prevail, whereas at distances higher than 10 nm, photon emission by donor would be dominant. Therefore, FRET occurs only in the near field, which is in the range of 1–10 nm [11,12]. Often FRET is envisioned as a phenomenon occurring between two spectroscopically different fluorophores, also termed heteroFRET. However, FRET can also take place between spectroscopically identical fluorophores under the condition that they have a small Stokes shift, *i.e.*, small separation between excitation and emission spectral peaks. Energy transfer between like fluorophores is known as homoFRET. When the two molecules in close proximity are fluorescent species, the apparent changes that would occur due to FRET are reflected in the spectroscopic properties of these fluorochromes, like fluorescence intensity, fluorescence lifetime, quantum efficiency and anisotropy [13,14]. FRET is intrinsically sensitive to molecular distance providing both quantitative and qualitative information leading to its widespread use in various disciplines of science. In fact, FRET is perfectly suitable for determining the large array of dynamic molecular events, including conformational change in macromolecules, *cis*- or *trans*- association/or assembly in macromolecules *etc.*, regulating physiological events both under *in vitro* and *in vivo* conditions [10]. The Jablonski diagram represents the simplest explanation of the occurrence of FRET in terms of donor/acceptor excitation and emission (Figure 1). We aim to introduce FRET techniques to the biologists or bio (medical) researchers who can hugely benefit from FRET applications. Therefore, this review is not a comprehensive report on FRET; rather it entails the phenomenological description of the mechanism of FRET, highlights advantages and limitations and the type of information that can be gained from FRET by using various methodologies, and presents several examples of FRET applications in membrane biology.

Förster theory states that the efficiency of energy transfer (E) is a function of the inverse sixth power of the distance separating the two interacting molecules and “E” is expressed by the following equation:

(1) $$\text{E}=\frac{R_{0}^{6}}{R^{6}+\;R_{0}^{6}}$$

In the equation above, “*R*” indicates the actual distance between donor and acceptor whereas “*R*_0_” represents the Förster distance, which is a characteristic distance at which the probability of energy transfer is 50%. Though, it is clear from Equation (1) that “E” is highly dependent on the magnitude of “*R*”, there are also other factors (see Equation (2)) that influence “*R*_0_” and correspond to the spectroscopic features of fluorescent donor and/or acceptor and their spatial orientation which can significantly affect energy transfer.

(2) $$R_{0}=0.02108\;{\left[\kappa^{2}\times \Phi_{D}\times n^{-4}\times J\right]}^{\frac{1}{6}}\text{ nm}$$

(3) $$\kappa^{2}={\left({\text{cosθ}}_{\text{R}}-3{\text{cosθ}}_{\text{D}}{\text{ cosθ}}_{\text{A}}\right)}^{2}$$

(4) $$J=\overset{\infty }{\underset{0}{\int }}{\text{F}}_{\text{D}}\left(\text{λ}\right){\text{ε}}_{\text{A}}\left(\text{λ}\right){\text{λ}}^{4}\text{dλ}$$

**Figure 2:**
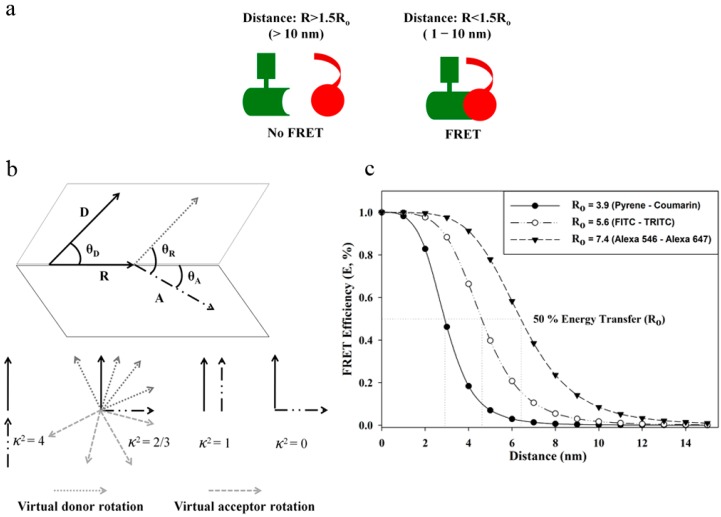
(**a**) A schematic representation of FRET between two molecules; (**b**) The orientation of emission dipole moment of donor and absorption dipole moment of acceptor is illustrated in this figure. “R” is the distance between the centers of donor and acceptor. θ_D_ is the angle between the transition dipole moment of donor and the line joining the two dyes while for the acceptor this angle is indicated as θ_A_. The angle between the donor and acceptor dipole moments is θ_R_. The possible virtual donor and acceptor fluorophore orientations are also presented in this figure, however, in reality the vectors are not exactly as depicted rather are random and can point in any direction of space; (**c**) Dependence of energy transfer on distance: A graph is presented here showing FRET efficiency (E) for three FRET-pairs with *R*_0_ values 3.9 nm (pyrene and coumarin), 5.6 nm (fluorescein isothiocyanate (FITC) and tetramethylrhodamine (TRITC)), and 7.4 nm (Alexa Fluor 546 (Alexa 546) and Alexa Fluor 647 (Alexa 647)) [18]. It is obvious that with the increase in *R*_0_, the sharp rise or fall of the graph at the end of the curves is reduced, allowing the feasibility to monitor changes in “E” at that range.

In the above equations, “*ĸ*^2^” is the orientation factor describing the relative orientation between the dipoles of the donor emission and the acceptor absorption, “*Φ_D_*” is the fluorescence quantum yield of the donor in the absence of the acceptor, “*n*” is the refractive index of the medium, and “*J*” is the extent of overlap between the donor emission and acceptor absorption spectra. In Equation (3), ${\text{ θ}}_{\text{R }}$ is the angle between the “D” and “A” dipoles whereas ${\text{θ}}_{\text{D}}{\text{ and θ}}_{\text{A}}$ are the angles the “D” and “A” dipoles, respectively, subtend with the line connecting the donor-acceptor dipole origins (Figure 2b). In Equation (4), “λ” is the wavelength and ${\text{ε}}_{\text{A}}\left(\text{λ}\right)$ and ${\text{F}}_{\text{D}}\left(\text{λ}\right)$ are the molar absorption coefficient of the acceptor and the normalized fluorescence emission of the donor at wavelength “λ”.

In practice, considering the use of fluorescent probes, the following set of conditions must be fulfilled in order to observe FRET: (I) The emission spectrum of the donor must overlap with the absorption spectrum of the acceptor. For a given FRET-pair, the larger the spectral overlap, the higher the Förster distance [15]; (II) The donor must have a high quantum yield; (III) The donor emission and acceptor absorption dipole moments must be oriented in favorable directions, which is numerically characterized by the orientation factor, *ĸ*^2^, ranging from 0–4. To observe FRET, *ĸ*^2^ should not be too small. FRET efficiency is the highest when the two vectors are parallel (*ĸ*^2^ = 4), whereas, FRET efficiency decreases with the increase in the angle between the two vectors. In fact, FRET efficiency is zero when the two vectors are in perpendicular position even in a case when two fluorescent probes are within FRET distance (*ĸ*^2^ = 0). For most biological applications considering the use of organic fluorophores, *ĸ*^2^ is usually taken as 2/3, which is an isotropic dynamic averaging, assuming that both donor and acceptor fluorophores can acquire all possible random orientations during the donor’s lifetime (Figure 2b) [16,17]. Furthermore, *ĸ*^2^ is also influenced by the nature of the microenvironment where donors and acceptors are suspended; (IV) Most importantly, the donor and acceptor must be close, but not too close to induce contact based quenching. Usually, the distance between 1 and 10 nm is reasonable (Figure 2a).

## 3. FRET: An Index for Sub-10 Nanometer Distances

The efficiency of FRET has a strong dependence on the Förster distance (*R*_0_) and on the physical distance separating the donor and acceptor species (*R*). *R*_0_ is a characteristic feature of each donor and acceptor FRET-pair and can be estimated based on Equation (2). It is generally in the range of 4–8 nm. *R*_0_ values for FRET-pairs can be found in the literature for both organic dyes [18] and fluorescent proteins [18,19]. It is obvious from the Figure 2c that a FRET-pair with a larger *R*_0_, e.g., *R*_0_ of Alexa 546-Alexa647 in comparison with pyrene-coumarin, accommodates a wider range of measurable distance for “E” thus providing possibilities to determine small-scale changes in distances more effectively. In fact, “E” shows saturation at a distance below 0.5 *R*_0_ (E sharply attains 1) and above 2 *R*_0_ (E dwindles to 0 rapidly). Thus, distances in the range of ~0.5 *R*_0_–~2 *R*_0_ can usually be measured by FRET. Beyond these distance limits, we can only state that the distances are below 0.5 *R*_0_ or beyond 2 *R*_0_ [11]. The critical working distance of FRET also matches the dimension of many biological molecules, such as the size of proteins (a 30 kDa globular protein has a diameter of ~3 nm [19]), lipids and nucleotides, the distance between two interacting macromolecules or sites on multi-subunit proteins *etc.* FRET is therefore perfectly suitable for biological research resulting in the description of FRET as a “spectroscopic ruler” to probe intermolecular distances. The choice of a FRET-pair, however, depends on the type of biological questions and the available instrument for FRET studies. The spatial resolution of the conventional optical microscope is limited by diffraction to ~250 nm laterally, which is orders of magnitude larger than the average size of a protein molecule ranging within a few nanometers. This makes it difficult to predict whether the two molecules in the image obtained by traditional microscopes are in interaction or not. In such cases, exploitation of FRET increases the accuracy of co-localization of the molecules within the diffraction-limited spots. This provides a good contrast mechanism, and occurrence of FRET between two molecules is proof of potential molecular proximity.

## 4. Lighting up Molecules for FRET

Essentially, a prerequisite for FRET is to be able to visualize molecules. Often, with some exceptions, biological molecules are not self-fluorescent. Therefore, tagging of target molecules with fluorescent markers is required. There are three popular approaches which can render the molecules of interest fluorescent: (1) An approach based on fluorescent affinity reagents prepared by conjugating fluorophores to affinity probes [20] (2) An approach based on fluorescent protein (FP) requiring fusion of DNA of target protein and fluorescent protein [21] and (3) An approach based on bioorthogonal chemistry for labeling proteins or an *in vivo* labeling approach in which a target protein is fused with a tag making it amenable for chemical labeling in living cells [22].

### 4.1. An Approach Based on Fluorescent Affinity Reagents

Antibodies are the most widely used affinity reagents in biological research owing to their high affinity and exceptional specificity towards the target molecule. They are also easy to generate, virtually against any known molecules, with the well-established hybridoma technology [23]. Fluorophore conjugated antibodies are popular as a probe for FRET or cellular imaging traditionally. Derivatives of organic fluorophores having functional groups with reactivity toward relevant side-chain groups, such as amines and sulfhydryls, in a protein are easily available. The literature also abounds with numerous straightforward bioconjugation protocols making preparation of fluorescent antibodies a relatively simple task nowadays [20,24]. However, the most widely used methods for bioconjugation, involving amine targeting, is not site-specific and, rather, is random in nature due to the abundance of amines in proteins [20,24], a condition which is not completely ideal for FRET measurements. Additionally, despite the presence of a large number of commercial fluorophores with emission ranges extending from the UV to the infrared spectrum [25], their suitability for FRET investigations is not easy to judge because of the difficulty in obtaining complete information about their photophysical features. Nonetheless, data about several fluorophore FRET-pairs in the literature are of great help in choosing the right fluorophores though it restricts us to only limited sets of few good FRET-pairs for studying membrane proteins [15,25,26]. Most of our FRET studies on membrane proteins were based on fluorophore-antibody conjugates. We have demonstrated earlier that fluorescent primary antibodies are preferred for FRET measurements. However, indirect labeling using secondary fluorescent antibodies can also be used to detect primary non-fluorescent antibodies. Although, such a scheme would lead to a decrease in the FRET efficiency between the probed proteins because of the greater separation of donors and acceptors as a result of the increase in the size of the antibody labeling complex. We also cannot rule out the possibility of detecting FRET between two proteins at distances greater than 10 nm. The use of polyclonal secondary antibodies can complicate the interpretation of FRET as well [15,27]. Antibodies with their large-size, molecular weight of ~150 kDa, and bivalent binding properties can also pose other problems. Since the latter circumstance can induce artificial clustering, there is a growing interest in alternative affinity reagents like aptamers—most often oligonucleotides [28], affibodies [23], synthetic engineered antibodies or phage-display antibodies [28,29] and single-domain antibodies (e.g., nanobodies engineered from heavy-chain camelid antibodies) [30] with target specificity equaling or exceeding that of conventional IgGs. Labeling of intracellular antigens with fluorescent antibodies also requires cell fixation and permeabilization. Fixation itself can alter the geometry and distribution pattern of molecules inside the cell [31] and can also damage or mask the antigenic structure leading to abrogation of antibody binding [32]. Overall, fluorescent affinity reagents have facilitated *in vitro* studies on protein structure and protein–protein interaction; however, it has not been successful in addressing questions about the real-time dynamics of molecules in living cell, which is important for studying weak or transient molecular interactions that might occur in natural cellular milieu.

### 4.2. An Approach Based on Fluorescent Proteins

The possibility of recombinantly fusing the target molecule with a fluorescent tag augmented the interest in FRET applications and also made it possible to follow biological processes in time in different cellular compartments in a non-invasive manner. Among the most popular tags are green fluorescent proteins (GFPs) and its derivatives. Like organic fluorophores, now we have a palette of different colored FPs, spanning the entire visible spectrum, as a result of GFP engineering and newer discoveries from corals and several unrelated species including crustaceans and lancelets. The spectrum also includes the modern red fluorescent proteins (RFPs) with larger Stokes shift and with emission maxima exceeding 560 nm [33,34,35,36]. In fact, the library of FPs offers a huge collection of donor-acceptor pairs suitable for FRET experiments [35]. However, CFP (cyan) and YFP (yellow) is still the most widely used donor-acceptor pair [37]. Recently, a combination of Clover and mRuby2, emitting green and red fluorescence, respectively, was found to increase the dynamic range and detection sensitivity of FRET in comparison with CFP-YFP pair [38]. Post-2007, several new FP variants have emerged with better photophysics compared to GFPs and RFPs [35]; therefore, quantitative examination of FPs suitable for FRET measurements in live cell imaging is an avenue that is open for exploration. In contrast to the approach based on fluorescent affinity reagents, FPs offer the possibility for site-specific conjugation to molecules and have revolutionized life science studies. However, FP fusion proteins are not free of problems. The significant molecular mass of FPs, e.g., 27 kDa (240 aa) for GFP, might alter expression, folding and distribution of the target protein and can impair protein function [39,40,41]. Most FPs show some tendency of self-association, especially when they are fused to other proteins; thus, they may induce artificial clustering. In fact, the self-association behavior of fluorescent proteins in physiological conditions might be completely different [40,42]. Therefore, the behavior of fusion proteins may not be the exact reflection of that of endogenous proteins and the requirement of ectopic expression further complicates it. FPs also show tremendous heterogeneity in maturation, sensitivity to pH, ionic strength, photobleaching kinetics [25,36,41] and possibly their behavior in various cell types [43]. The spectroscopic features of FPs, such as brightness and photostability, are relatively inferior to that of organic fluorophores [36,43]. Further, switching to another color requires either another plasmid vector having target protein fused to another FP or recloning, making exchange of colorsa difficult process.

### 4.3. An Approach Based on Bioorthogonal Chemistry

To address some of the issues with FPs, bioorthogonal chemistry based labeling approach was developed. The idea here is to express a tag that would allow site-specific labeling in the target protein with luxury for swapping of fluorophores with known photophysical features. Several new methods based on this concept have emerged recently which include expression of tags that could bind specific ligand, enzyme mediated ligation of tag with fluorophore substrate, and unnatural amino acid incorporation into proteins for specific chemical labeling. A detailed description of these methodologies is not the subject of this review but some excellent reviews have been published earlier [22,43,44]. From our perspective, we find SNAP and CLIP technology to be relatively more flexible for FRET measurements than the other bioorthogonal approaches, though, the combination of bioorthogonal approaches can work successfully. Both SNAP and CLIP proteins are derivatives of human DNA repair protein O_6_-alkylguanine-DNA alkyltransferase (hAGT) performing self-attachment but with preferential reactivity towards O_6_-alkylylguanine or O_6_-benzylguanine and O_2_-benzylcytosine derivatives as substrates, respectively. Because of these orthogonal substrate specificities, SNAP- and CLIP-tagged fusion proteins can be simultaneously labeled with different molecular probes bearing fluorophores. SNAP- and CLIP-tagged proteins are similar in size (~20 kDa) to FPs; however, they confer an important advantage with respect to the choice in organic fluorophore that can be used to label the tagged fusion proteins for various types of FRET experiments [45]. In comparison with FPs, biological research with SNAP- and CLIP-tagged proteins is still in its infancy; therefore, the possible effects of these tags on the expression, maturation, localization and function of the target proteins are uncertain. However, it is known that the life-span of hAGT is dramatically reduced and is targeted for ubiquitin degradation upon forming a complex with its substrate [46]; therefore, SNAP- and CLIP-tagged fused proteins may have reduced half-life in comparison with their endogenous counterparts. Nonetheless, in a recent study, it was demonstrated that SNAP-tagged fusion proteins retained the normal distribution features of wild type protein whereas several FPs induced artifacts due to clustering of the fused protein [40]. Endogenous hAGT is also known to react with SNAP substrates, though less efficiently, which would lead to off-target background noise. However, endogenous hAGT is comparatively very low in most cell lines and can be avoided when hAGT deficient cell lines are used [45]. In contrast to FPs, maturation and fixation of SNAP- and CLIP-fused proteins do not seem to be an issue either because fluorophores with known photochemical features are used [25,36,47]. Importantly, these methods can be used in conjunction with tetracysteine (CCXBCC, where X and B can be any amino acid except cysteine), tetraserine (SSPGSS) or Halotag based approaches amongst others providing multiple efficient ways for multicolor labeling [22,25,43,44]. Thus, bioorthogonal labeling of SNAP- and CLIP-tagged proteins seems flexible, non-invasive and the most suitable strategy for FRET experiments. However, any FRET experiment should be cross-checked with an alternative strategy for confirmation of the findings.

## 5. Is Your Instrument FRET Friendly?

In principle, any instrument capable of recording fluorescence emission can be used for measuring FRET as long as suitable fluorophores are available and the corresponding filters and detectors are present in that system. The theoretical foundation of FRET was laid out on the basis of collision experiments of several metallic elements in the vapor phase using a spectroscope [4]. Therefore, early FRET experiments were mainly performed using spectrofluorometry [6,48,49] then it slowly progressed to flow cytometry [49,50,51] and various microscopies [21,49,52,53,54] or lately to laser scanning cytometry [55,56,57]. However, generally the choice of instruments for FRET measurements is driven by biological goals because each of these instruments can deliver specific types of answers. Of course, the most versatile of all the instruments is the microscope; therefore, many FRET approaches are devised exploiting the capabilities of microscopy to decipher the spectroscopic information of fluorophores. Thomas Jovin and colleagues proposed some new microscopic approaches and also prepared a catalog of FRET techniques in 2003 [58]. Likewise, critical evaluation of many of the widely used FRET methods in spectrofluorometry, flow cytometry and microscopy has also been performed in [59]. Therefore, our goal in this section is just to describe the merits and demerits associated with each of these widely used instruments for FRET. FRET crawled its way into biology through physics and chemistry. Therefore, it is no surprise that the fluorometer was the most commonly used instruments for measuring FRET in the early days, even today in many places, because of its easy accessibility. Donor quenching and acceptor sensitization based FRET experiments are primarily performed in spectrofluorometry. Fluorescence signals are collected from the cell suspension either in the cuvette or from the cells fixed on slides. Therefore, the acquired signals are an average from thousands of cells. These are good for statistical accuracy, but possible heterogeneity among cells in the sample is hidden. Measurements from fluorometer thus are blind-folded from the details of each cell and they provide population averages of parameters. Since samples are measured in solution, the presence of dead cells or cellular debris (which can often be far brighter), free fluorophores (especially with low-affinity fluorophore labels), and cellular autofluorescence can affect the measurement significantly [50,60]. The contribution of the aforementioned factors in the measurement accentuates the notion that performing FRET for lowly expressed proteins is almost impossible or unreliable due to distortion in signals with fluorometry that in many cases would require cell-by-cell correction for autofluorescence [27]. Because of the measurement procedure, sample should be prepared very carefully, for instance, proper washing should be performed and contribution of free fluorophores to the fluorescence intensity should be minimized. Microscopy and flow cytometry can overcome the above limitations of spectrofluorometry. However, relatively very few cells can be investigated in microscopy compromising its statistical reliability [50]. Microscopy is more suitable for real-time visualization of kinetic molecular events capturing microsecond or nanosecond cellular changes in living cells and providing crucial information regarding the functioning of biological molecules [61]. FRET is commonly used with wide-field microscope but has been successfully implemented in confocal, multi-/two-photon, fluorescence lifetime and fluorescence polarization microscopes, each with their own distinct advantages. For example, wide-field microscope collects the emission signals from both above and below the focal plane, reducing the quality of the image, whereas, confocal microscopes can reject these out-of-focus signals giving sharper images. In combination with fluorescence lifetime or anisotropy, the sensitivity of FRET approach is enhanced providing a spatial map and temporal information pixel-by-pixel in different contrast patterns [13,61,62]. Therefore, microscopy is more suitable for studying dynamic molecular events and heterogeneity in or within a cell [63,64]. Regardless of the benefits of microscopes for FRET measurements, the underlying disadvantage of all microscopes is that they require specific training on the proper usage of these microscopes and the ability to record high-quality fluorescence images in the absence of any artifacts. A flow cytometer on the other hand can measure thousands of cells within a minute. Therefore, the results are more robust and reliable than those from microscopes statistically; however, information can only be obtained on a cell-by-cell basis. Nonetheless, it allows categorization of a population of cells from the same sample and is capable of elucidating the differences in “E” in correlation with any other cellular parameters. Importantly, what flow cytometer can achieve is the possibility of sorting the cells based on cellular heterogeneities and energy transfer [65,66]. However, the disadvantage of using flow cytometer is the nature of measurement of cells. Cellular suspension is necessary which means detachment of cells from their substrate should be performed. Removal of cells from their natural environment either mechanically or by enzymatic treatment can result in changes in cellular parameters. Furthermore, flow cytometry fails to provide information regarding heterogeneities within the cell and about the time-course of cellular responses at single cell level, the attribute at which microscopy excels [60]. The superiority in statistics offered by flow-cytometric measurements thus compensate for the lack of subcellular information that can be obtained in microscopy [15,60]. High-throughput imaging technologies can combine the complementary features of both microscopy and flow cytometry. We have recently demonstrated the applicability of FRET with a laser scanning cytometer (LSC), LSC-FRET, yielding FRET efficiencies comparable to those obtained from a flow cytometer or a confocal microscope [55]. One major advantage of LSC is that spatial resolution and information about subcellular structures can be generated under native *in situ* conditions with decent statistics. LSC yields information on a pixel-by-pixel and cell-by-cell basis with the capability for temporal cellular response analysis in cells. Thus, LSC advances the features of flow cytometry with a small sacrifice in the number of cells that could be analyzed in the specific time-frame but with the incorporation of advantages of confocal microscopy. Likewise, a high-throughput FRET technique based on total internal reflection fluorescence microscopy (TIRFM) was also published a few years ago [67]. The inherent advantage of FRET-TIRFM is similar to that of LSC-FRET. However, it harbors few additional benefits specific to the use of evanescent wave for illumination of samples. Firstly, evanescent wave generates low autofluorescence because TIRFM excitation is limited to a thin layer (~100 nm, mainly plasma membrane). Secondly, in TIRFM fluorophores located closer to the plasma membrane are preferentially excited; therefore, FRET-TIRFM is efficient in removing non-membrane fluorescence. In fact, TIRFM is said to be far superior to confocal microscopes at detecting plasma membrane fluorescence [67]. Thus, it is optimal for high-throughput FRET related to membrane proteins.

In the paragraphs below, we will discuss several methods that are generally used in biological systems for measuring FRET and subsequently we will also elaborate on the various applications of FRET based techniques, especially on our contribution towards developing and applying FRET based methods for studying membrane proteins.

## 6. Methods for Measuring FRET

**Figure 3:**
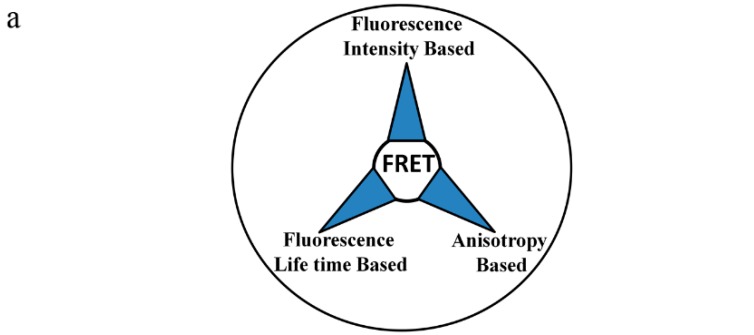

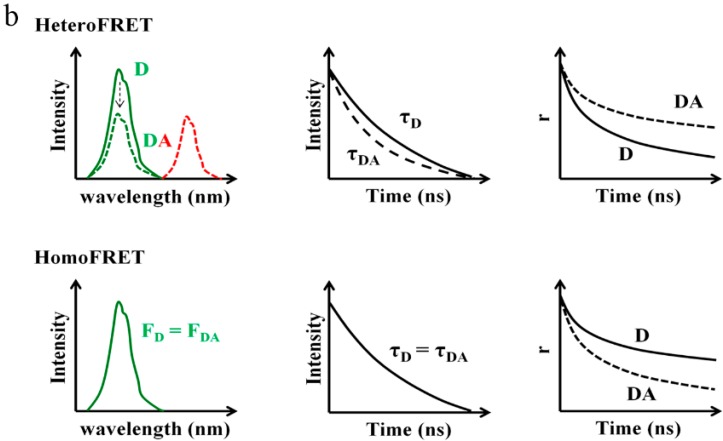
(**a**) Schematic representation of types of FRET measurements based on photophysical features; and (**b**) This figure illustrates the effect of heteroFRET and homoFRET on fluorescence intensity (**left** panels), fluorescence lifetime (**middle** panels) and fluorescence anisotropy (**right** panels). Here, “D” and “DA” quoted symbols, including the ones in the subscript, represent donor only and FRET samples. In heteroFRET, donors and acceptors are fluorophores with different spectroscopic features. In the upper graph in the left panel, the emission of donor and FRET samples on excitation by the donor excitation wavelength are depicted for simplicity. The green and red curves correspond to the donor and acceptor emission spectra, respectively, in the left panels. In the lower graph in the left panel, there is only a green curve since both the donor and the acceptor are spectroscopically identical. In the middle and right panels, only the time-dependent donor emission (middle) and anisotropy (right) are shown in the absence and presence of the acceptor. The only manifestation of homoFRET is the decrease in anisotropy and no change in fluorescence intensity or fluorescence lifetime of the donor fluorophore.

Fluorescence has many spectroscopic dimensions that can be sensitively monitored and fortunately, the manifestation of FRET is the alteration of these spectroscopic features. Therefore, a multitude of techniques can be employed to measure FRET. Several excellent reviews have been published earlier [11,13,58,68,69,70,71] where extensive theoretical details are underlined for quantitative analysis of FRET. Types of FRET and the effect of FRET on the spectroscopic features of fluorophores are schematized in Figure 3. The most notable FRET measurements are based on the following three approaches: (1) Fluorescence intensity based approach; (2) fluorescence lifetime based approach; and (3) fluorescence anisotropy based approach. We have played a seminal role in the development of a ratiometric flow-cytometric FRET (FCET); therefore, FCET will be discussed extensively here, whereas, other FRET methods will be presented through a few mathematical expressions for easy understanding.

### 6.1. Fluorescence Intensity Based Approach

#### 6.1.1. Donor Quenching Method

This is the most straight-forward and the easiest method for quick measurement of FRET. It requires an inspection of donor fluorescence in singly (donor only) and doubly (donor-acceptor) labeled samples. The consequence of FRET is the decrease in the fluorescence intensity of the donor in the doubly labeled sample in comparison with the intensity of the donor from the donor only sample. However, this method is error-prone because any quenching observed in the donor fluorescence intensity is assumed to be due to the presence of acceptors. Since the donor intensity is measured in two different samples (donor-labeled and donor-acceptor double-labeled), any difference in the expression level of the labeled antigens, alterations in antibody affinity and spectral cross-talk between the acceptor and donor fluorescence can lead to a difference between the donor intensity in the two samples. Obtaining the mean fluorescence intensity from a large population of cells minimizes the variation in fluorescence at the individual cell level. However, this also prevents donor quenching FRET from providing FRET information on a cell-by-cell basis, thus only mean FRET efficiency representative of a population of cells can be obtained. Therefore, this approach is mainly suitable for flow cytometry or spectrofluorometry based FRET studies [10,15,19]. Controls especially to identify competition in antibodies used for labeling the proteins should also be considered. One should make sure that the antibodies do not influence the binding of each other. If any, correction should also be performed for acceptor spill-over in the donor channel [15,19]. Intensities are measured by exciting the sample at the absorption peak of the donor and detecting fluorescence at the emission peak of the donor.

Assuming “*F*_D_” is the fluorescence intensity of the donor sample and “*F*_DA_” is the fluorescence intensity of the donor and acceptor labeled sample, energy transfer is calculated with the following equation:

(5) $$\text{E}=1-\;\frac{F_{\text{DA}}}{F_{\text{D}}}$$

Both *F*_D_ and *F*_DA_ have to be background-corrected, *i.e*., the fluorescence of unlabeled cells has to be subtracted.

#### 6.1.2. Acceptor Photobleaching Method

This is mainly a microscopy-based method. Importantly, FRET is estimated from information obtained after imaging a single sample. The general principle is to compare the fluorescence intensity of the donor before and after photodestruction of acceptor species. In the case of occurrence of FRET, there is an increase in the fluorescence intensity of the donor (donor dequenching) after bleaching of acceptors. Since high-intensity laser is used for bleaching of acceptors and acceptor molecule is irreversibly switched off, but remains physically connected to the donor even after bleaching, this approach has a few drawbacks. For example, generation of dark acceptors (non-fluorescent acceptor products, but capable of donor quenching), incomplete photobleaching of acceptor molecules and bleaching of donor species are still possible. Likewise, photobleaching of acceptors can also yield acceptor degradation products with an emission profile similar to that of donor molecules. Importantly, photodestruction of fluorophores means repeated measurements of the same sample is not possible precluding real time information on macromolecules [59,71] although identification of photoswitchable dyes [72] and fluorescent protein [73] has offered possibilities for dynamic measurements. Photosensitive acceptors and photostable donors are perfect for the acceptor photobleaching technique. Importantly, precaution should be taken to avoid movement of cells during photobleaching. The mathematical expression for the calculation of energy transfer (E) is analogous to Equation (5) except that *F*_DA_ is replaced with the fluorescence of donor before acceptor bleaching and *F*_D_ is substituted by the fluorescence of the donor after photobleaching.

#### 6.1.3. Sensitized Acceptor Excitation Method

FRET measurement based on quantification of sensitized emission of the acceptor is the most reliable among all intensity-based methods. Sensitized emission of acceptor is the amount of acceptor emission in the FRET channel due to resonance transfer of excitation energy from donor to acceptor [59]. Simultaneous measurement of individual fluorescence emissions from donor and acceptor from the same sample, in comparison with multi-samples, excludes problems related to variation, such as changes in donor density or fluorescence quantum yield [59,68]. Nonetheless, FRET estimations are more accurate and easier to perform in a case when donor and acceptor emissions are well separated. Otherwise, this method invites the introduction of several correction factors related to fluorophore cross-talk, *i.e*., the reciprocal excitation of donor and acceptors at the excitation wavelength of the other dye, and bleed-through of fluorescence emission of the donor and the acceptor to detection channel corresponding to the other dye. Independent control samples of donor and acceptor can help compensate the issues related to the above problems. Numerous methods have evolved with a goal of quantifying sensitized acceptor signal. Although quantitative approaches determining the FRET efficiency rigorously are preferred [53], semi-quantitative method providing uncalibrated FRET indices with dubious theoretical background also abound in the literature [59]. FRET indices are instrument dependent relative values designed according to the aims of studies. They are qualitative in nature, however, some of them seem to be more sensitive and consistent in cases where FRET efficiency based methods tend to suffer, for instance, when the ratio of donor to acceptor is lower than 1 [59]. With its simple mathematical framework, these approaches would seem rather attractive to biologists who are more concerned about learning the possibility of interactions between two macromolecules in a simple “yes” or “no” format or knowing the consequence of a biological response in the association of proteins in relative terms. Based on the literature, methods for measurement of sensitized emission can be categorized into three groups with the basic difference being the process of analyzing FRET signals: (1) Two-channel emission or excitation ratio measurement; (2) three-channel emission measurement; and (3) spectral analysis for FRET.

##### Two-Channel Emission or Excitation Ratio Measurement

Two channel emission ratio measurement has been applied a lot both in microscopy [74,75] and flow cytometry [76,77] as sensors of protein–protein interactions. Basically, the practice is to illuminate the doubly (donor and acceptor) labeled sample with the donor excitation wavelength, then, collect the signals in both donor and FRET channels, *i.e*., in the wavelength range corresponding to the emission peak of the donor and acceptor, respectively. A FRET index defined as the ratio of fluorescence intensities in the FRET and donor channels are widely used owing to the fact that the ratio is fairly consistent [19,52,78]. An alternative two-channel excitation ratio measurement has also been described before, where measurements at the emission wavelength of acceptor were taken upon consecutive excitation of the FRET sample with donor (FRET channel) and acceptor (acceptor channel) wavelengths. In this case, a parameter proportional to the FRET efficiency is expressed as the ratio of fluorescence in the FRET channel to the fluorescence in the acceptor channel [78,79]. Primarily, the above methods are ignorant to multiple cross-talks and bleed-through features of fluorophores such as direct excitation of the acceptor at the donor absorption wavelength. However, these methods are very useful as long as the ratio of the concentrations of donors and acceptors is constant such as in intramolecular FRET studies [52,54] involving FRET-sensors.

##### Three-Channel Emission Measurement

Three-channel emission measurement requires collection of three independent signals from the same sample. These signals differ either in the wavelength of excitation or in a spectral range of detectors for recordings. In fact, this method is similar to the two-channel measurement with an additional third channel making rigorous deductions of non-FRET signals possible. Numerous studies have been published representing such a set-up with varying level of stringency with regards to cross-talk or bleed-through corrections [59]. For three-channel measurement, assessment of FRET has been demonstrated both in terms of FRET indices [52,53,80,81] and FRET efficiencies [15,54,68,69,82,83]. Measurements require collection of signals for donor alone, acceptor alone and doubly (donor-and-acceptor) labeled samples under the same circumstances. Below, we will describe three of the most popular FRET index based three-channel measurements with FRET terminologies used by the respective authors:

(a) Corrected FRET (*F*^c^) method: This method was introduced by Youvan *et al*., for epifluorescence microscope [80]. They simply generated a FRET image corrected for fluorescence from background and bleed-through. However, the contribution of reciprocal cross-talk excitation in donor and acceptor channels, which were minimal in their case, was not considered during the calculation. Similarly, the method does not perform normalization for concentration of donors and acceptors. Therefore, it inherently suffers from the issues related to variability in fluorophore concentration. In fact, even at the same FRET efficiency, the FRET signal is different for samples in which various concentrations of donors and acceptors are used. Thus, it is suitable under conditions when the donor to acceptor concentration is constant or known beforehand. The corrected FRET was expressed in the following form:

(6) $$F^{\text{c}}=\;F_{\text{f}}\;–[(F_{\text{d}}/D_{\text{d}})\times {\text{ D}}_{\text{f}}]\;–\;[(F_{\text{a}}/A_{\text{a}})\times A_{\text{f}}]$$

In Equation (6), *F*, *D* and *A* represent FRET, Donor and Acceptor channels, respectively, whereas subscripts “f”, “d” and “a” represent FRET, donor and acceptor samples, respectively. The spectral bleed-through for donor (*F*_d_/*D*_d_) and acceptor (*F*_a_/*A*_a_) are calculated from donor only and acceptor only samples, respectively. It is also assumed that the images were background subtracted in the above equation.

(b) FRET net (FRETN) method: Gordon *et al*. presented a FRETN method to overcome the underlying problem with the *F*^c^ method. *F*^c^ is linearly proportional to the concentration of fluorophores; therefore, they proposed that Equation (6) should be additionally normalized by the product of donor and acceptor signals [54]. This new method, however, overcompensates by dividing the *F*^c^ value with both donor and acceptor intensities. Therefore, FRET values flatten out at higher donor and acceptor intensities whereas it is fairly sensitive at low donor and acceptor intensities. Thus, this method generates FRET values with high standard error (80%) affected by concentrations of donors and acceptors [84].

(7) $$\text{FRETN }=\;F^{\text{c}}/\;G\times \;D_{\text{f}}\times A_{\text{f}}$$

The notations in Equation (7) are similar to that of Equation (6). A constant “*G*” is a parameter which relates the loss of donor signal to the increase in acceptor signal as a result of FRET (please refer to Equations (15) and (16) for “*G*” which is equivalent to “α” in the section below).

(c) Normalized FRET (N_FRET_) method: In order to reduce the inconsistency of the FRETN method, Xia et al introduced the normalization procedure for F^c^ with the product of the square root of donor and acceptor signals [84]. This renders FRET independent of local concentration of fluorophore. However, N_FRET_ is still not linear with changes in E values and fractional occupancy; therefore, it is not adequate for stoichiometric measurements of binding interactions [85].

(8) $${\text{N}}_{\text{FRET}}\;=\;F^{\text{c}}/\sqrt{D_{\text{f}}\times A_{\text{f}}}$$

Several other methods have also been published where the basis of normalization of FRET value is with acceptor concentration [81,86].

Experimental results obtained using FRET indices from different instruments are not comparable because FRET indices depend on system parameters such as excitation intensities and detection efficiencies of the instrument [21]. Therefore, it is more relevant to express FRET through an instrument-independent, but quantitative parameter such as FRET efficiency. Assuming that the FRET-pair is red-shifted, which minimizes autofluorescence [27], and the contribution of background is negligible, FRET efficiency can be easily computed by solving a set of three linear equations corresponding to the signals from a FRET sample. They are expressed as a function of unquenched donor (*I*_D_), the FRET efficiency (*E*) and intensity from the acceptor in the absence of FRET (I_A_). To maintain consistency, we are using similar terminologies as in our previous papers [15,68,69,82]. The following equations are based on FCET [15,68], although, we also implemented it in microscopy [69,70]. First, we would like to introduce the correction factors, which need singly labeled samples of donor and acceptor, so that it becomes easy to follow the equations. Correction factors result from spill-over and cross-excitation between donor and acceptor fluorophores, thus, are necessary for eliminating non-FRET signals. In a general case, four different “*S*” factors are used, namely *S*_1_, *S*_2_, *S*_3_ and *S*_4_.

*S*_1_ and *S*_3_ characterize the spill-over of donor intensity to the FRET (*I*_2_) and acceptor (*I*_3_) channels, respectively, and are determined using donor only labeled sample.

(9) $$\begin{array}{cc} S_{1}\;=\frac{I_{2}}{I_{1}}, & S_{3}\;=\frac{I_{3}}{I_{1}} \end{array}$$

*S*_2_ and *S*_4_ characterize the spill-over of acceptor intensity to the FRET (*I*_2_) and donor (*I*_1_) channels, respectively, and are determined using acceptor only labeled sample.

(10) $$\begin{array}{cc} S_{2}\;=\frac{I_{2}}{I_{3}}, & S_{4}\;=\frac{I_{1}}{I_{3}} \end{array}$$

where *I*_1_, *I*_2_ and *I*_3_ correspond to intensities measured in the donor, FRET and acceptor channels, respectively. The excitation (λ_ex_) and emission (λ_em_) wavelength for each of the intensities is defined in the parenthesis of Equations (11)–(13). For instance, the abbreviation in the symbol (λ_ex,D_; λ_em,D_) means excitation at the wavelength corresponding to the donor absorption band, and emission detected at the wavelength corresponding to the donor emission wavelength range. The uppercase letters “D” or “A” in the symbols represent donor and acceptor, respectively.

(11) $$I_{1}\left({\text{λ}}_{\text{ex},\text{D}};{\text{λ}}_{\text{em},\text{D}}\right)=I_{\text{D}}\left(1-\text{E}\right)+I_{\text{A}}\times S_{4}+I_{\text{D}}\times E\times \text{α}\times \frac{S_{4}}{S_{2}}$$

(12) $$I_{2}\left({\text{λ}}_{\text{ex},\text{D}};{\text{λ}}_{\text{em},\text{A}}\right)=I_{\text{D}}\left(1-E\right)\times S_{1}+I_{\text{A}}\times \;S_{2}+I_{\text{D}}\times E\times \text{α}$$

(13) $$I_{3}\left({\text{λ}}_{\text{ex},\text{A}};{\text{λ}}_{\text{em},\text{A}}\right)=I_{\text{D}}\left(1-E\right)\times S_{3}+I_{\text{A}}+I_{\text{D}}\times E\times \text{α}\times \frac{1}{S_{2}}\times \frac{{\text{ϵ}}_{{\text{λ}}_{\text{A}}}^{\text{D}}\;{\text{ϵ}}_{{\text{λ}}_{\text{D}}}^{\text{A}}\;}{{\text{ϵ}}_{{\text{λ}}_{\text{D}}}^{\text{D}}{\text{ϵ}}_{{\text{λ}}_{\text{A}}}^{\text{A}}}$$

Here, “ϵ” stands for molar absorption coefficient of “D” and “A” molecules shown by the upper indices at donor (${\text{λ}}_{\text{ex},\text{D}}$) or acceptor wavelengths (${\text{λ}}_{\text{ex},\text{A}}$). Often, *S*_3_, *S*_4_ and the molar absorption ratio, $\left(\frac{\epsilon_{{\text{λ}}_{\text{A}}}^{\text{D}}\;\epsilon_{{\text{λ}}_{\text{D}}}^{\text{A}}\;}{\epsilon_{{\text{λ}}_{\text{D}}}^{\text{D}}\epsilon_{{\text{λ}}_{\text{A}}}^{\text{A}}}\right)$, are negligible, as with the Cy3-Cy5 FRET-pair when measured on a FACSCalibur (BD Bioscience, San Jose, CA, USA). Thus, solving Equations (11)–(13) would yield “E” in the following form (see references [15,68] for the derivation):

(14) $$\text{E}=\frac{I_{2}-\;I_{1}S_{1}-\;I_{3}S_{2}}{\text{α }I_{1}+\;I_{2}-\;I_{1}S_{1}-\;I_{3}S_{2}}$$

It is also clear from the above set of equations that calculating “E” in ratiometric FRET requires determining a factor “α”, which has been widely used as “G” in microscopy, to correct for the differences in the quantum yield of the donor and acceptor and in the detection efficiencies of the donor in the donor channel and the acceptor in the FRET channel. “α” relates the loss of donor fluorescence to the sensitized emission of the acceptor.

Classically, “α” is expressed as in the equation below:

(15) $$\text{α}=\frac{Q_{\text{A }}{\text{η}}_{\text{A }}}{Q_{\text{D }}{\text{η}}_{\text{D}}}$$

where “*Q*_D_” and “*Q*_A_” are the fluorescence quantum yields of the donor and acceptor fluorophores, respectively, and “η_D_” and “η_A_” are the detection efficiencies of the donor in the donor channel and the acceptor in the FRET channel, respectively. Since both the quantum yield and the detection efficiencies are difficult to determine or calculate, numerous ways of calculating “α” factor have been reported previously though not without the challenges owing to the underlying variables. The interested readers are referred to the cited references for learning various approaches of determining “α” factor in microscopy [21,55,69,70,83,85] and flow cytometry [15,55,65,87]. One of the simplest approaches for calculating “α” is based on labeling two separate samples. One of the samples is labeled with a donor-tagged antibody and the other with an acceptor-tagged antibody. In such a case, “α” can be calculated according to the following equation:

(16) $$\text{α}=\;\frac{I_{2,\text{A }}}{I_{1,\text{D }}}\times \frac{B_{\text{D }}}{B_{\text{A }}}\times \frac{L_{\text{D }}\;}{L_{\text{A }}\;}\times \frac{\epsilon_{{\text{λ}}_{\text{D}}}^{\text{D}}}{\epsilon_{{\text{λ}}_{\text{D}}}^{\text{A}}}$$

where *I*_2,A_ and *I*_1,D_ are the intensity of the acceptor-labeled sample measured in the FRET channel and the intensity of the donor-labeled sample measured in the donor channel, respectively. Likewise, “*B*_D_” and “*B*_A_” are the mean number of epitopes labeled by the donor-conjugated and acceptor-conjugated antibodies, respectively, and “*L*_D_” and “*L*_A_” denote the labeling ratios (*i.e*., number of fluorophores/antibody) of the donor-conjugated and acceptor-conjugated antibodies, respectively. For the sake of simplicity, it is advisable to label the same epitope with the donor- and acceptor-conjugated antibodies, so that *B*_D_ = *B*_A_. Unfortunately, the method described above requires measuring a large number of cells, so that the mean intensities (*I*_2,A_ and *I*_1,D_) are reliably determined, which is difficult to achieve in microscopy. Alternatively, “α” can be calculated by labeling the same membrane protein with two non-competing antibodies binding to distinct epitopes but far enough to avoid any occurrence of FRET. One of the antibodies should be donor-tagged, while the other should be acceptor-conjugated. This approach ensures that the *B*_D_/*B*_A_ ratio is equal to 1 since the intensities *I*_2,A_ and *I*_1,D_ are measured on the same cells. Thus, “α” can be easily calculated from Equation (16) with *B*_D_/*B*_A_ = 1. If the requirement for no FRET cannot be met, the energy transfer taking place between the donor- and acceptor-labeled antibodies has to be taken into consideration [55,88].

Despite the use of such a systematic method for calculating “E”, satisfactory results are still difficult to obtain for proteins with low expression levels. Thus, in cases when the signal to noise ratio is very low, accurate FRET calculations require cell-by-cell correction for autofluorescence and selection of a FRET-pair with long emission wavelength. This improvement reduces the dispersion of FRET histograms and thereby improves the sensitivity of FRET analysis [15,27,69]. Likewise, we have also recently introduced an efficient method applicable in such cases called Maximum Likelihood Estimation (MLE) of FRET efficiency. The method is based on the assumption that photon detection by detectors follows Poissonian statistics [63]. We developed a computational tool applying the Poisson function to I_2_ intensity expressed as a function of I_1_ and I_3_ after solving Equations (11)–(13). The method thus predicts the joint probability of photon numbers received by the donor, FRET and acceptor channels. The presented algorithm assigns a single FRET efficiency based on the likelihood of all three measured intensities to each pixel. Therefore, outlier pixels with low probabilities for the determined FRET efficiency can be easily excluded from the analysis thus improving the accuracy of the calculation significantly. The only drawback is that MLE of FRET requires a dataset of at least 100 pixels, corresponding to region of 1 μm × 1 μm assuming a pixel size of 100 nm, for accurate determination of FRET efficiency; therefore, pixel-by-pixel documentation of “E” in an image is not possible. However, heterogeneity in spatial subsets due to biological variance can be explored when regions of interest are selected for analysis in the cell. With physiological settings, known to have low photon numbers due to weak expression of proteins and with abundant outer pixels of both biological and instrumental origins, we noted that MLE of FRET efficiency exceeds the performance of both pixel-by-pixel and total intensity based FRET approaches. The traditional method of calculating “E” suffers from distortion generated by detector noise, thus uncertainties prevail while calculating “E” for weakly expressed proteins. In fact, the FRET histogram would be wide and asymmetrical with large variance making “E” meaningless. Readers interested in the theoretical and mathematical background on this method can review our recently published paper [63]. The method was developed for confocal microscope; however, we do not see any reason why it cannot be adapted to flow cytometry as long as photon counting detectors are used.

##### Spectral Analysis for FRET

The idea of spectral analysis for FRET was borrowed from remote sensing and satellite imaging techniques [89]. The approach is to record a set of images in a series of wavelength bands, also referred to as lambda (λ) stacks. It is assumed that each fluorophore has its own specific spectral signature, which can be identified within the λ stack. With these signature reference spectra of the fluorophores and autofluorescence, the contribution of each fluorophore and autofluorescence in a mixed spectrum, as in a FRET sample, can be easily identified, even with a high degree of spectral and spatial overlap, using linear unmixing algorithms [90,91,92,93,94,95]. Taking advantage of this feature, several FRET approaches have been described; in fact, in many of the cases spectral imaging was just used as an addendum to the traditional FRET approaches to increase accuracy [90,92,93]. However, separating acceptor bleed-through from FRET signal is very difficult to obtain with linear unmixing because of their identical emission spectrum. This approach is primarily suitable for two-photon microscopy where judicious selection of excitation wavelength could be achieved easily thus circumventing acceptor cross-excitation [96]. Typically, the emission spectra of the donor and acceptor contain all the information regarding the concentration of fluorophores and the FRET efficiency [97]. Despite the possibility in increased sensitivity offered by spectral FRET measurement, the whole approach is complicated and requires special hardware for recording “λ” stacks. Additionally, the idea to distribute emission spectra in a series of spectral intervals to multiple detector channels also demands modification in instrumental settings (e.g., laser power, line averaging or pixel dwell time) for each of these channels potentially making each of the acquired images noisy [94]. Spectral FRET analysis is primarily used in microscopy; however, it is also feasible in spectrofluorometry [92,95] and spectral flow cytometry.

#### 6.1.4. Donor Photobleaching Method

Monitoring the photobleaching kinetics of donor in the presence or absence of acceptor also offers a simple approach to determine FRET. Photobleaching occurs from the excited state of a fluorophore. The stability of donors increases due to decrease in the availability of the excited state donor molecules when nearby acceptors are present due to FRET. Consequently, energy transfer decreases the rate of photobleaching and increases the bleaching time constant of the donor [57,82,98]. In contrast to the acceptor photobleaching technique, this approach requires a photolabile donor and photostable acceptor allowing determination of FRET efficiency [58,68,99]. This method also offers the advantage of being insensitive to expression density of proteins under investigation (since the kinetics of bleaching are measured which is assumed not to be influenced by the expression levels unless the expression level alters FRET), however, other environmental factors, like oxygenation, fluorophore concentration, temperature, *etc.,* can still influence donor photobleaching. The measurements are not self-controlled; therefore, mixing singly and doubly labeled cells and measuring them on the same slide sequentially or simultaneously for the bleaching kinetics would reduce errors due to the above factors. Since pixel-by-pixel bleaching time can differ due to molecular environment or other factors, it is considered to be more effective with wide-field microscopy instead of confocal laser scanning microscopy [68,100]. Donor photobleaching can be correlated to the FRET efficiency using the equation below:

(17) $$\text{E}=1-\frac{T_{\text{D}}}{T_{\text{DA}}}$$

In Equation (17), *T*_D_ and *T*_DA_ stands for the photobleaching time constant of donor in the absence and presence of acceptor, respectively. For calculating time constants, a sequence of images of donor from the corresponding samples are taken with donor-specific optical filter sets while bleaching the donor until it reaches the level of background. Since images are recorded at different times, the image stacks store the time-dependent fluorescence of the donor, or bleaching curve, which is fitted by the following exponential function resulting in the desired photobleaching constants [49,68,69,82].

(18) $$I_{\text{t}}=I_{0}{\text{e}}^{-\frac{t}{T}}+bg$$

where *I*_t_ is the time dependent donor fluorescence intensity, *I*_0_ is the donor intensity before bleaching, *t* is time, *T* is the bleaching time constant and *bg* is the background. In some cases, a single exponential function is not sufficient to achieve reasonable fits. In these cases a double exponential fit may be carried out [57].

### 6.2. Fluorescence Lifetime Based Approach

Fluorescence lifetime (τ) characterizes the time spent by fluorescent species at the excited state before exiting to the ground state by radiative and non**-**radiative mechanisms. Therefore, it is inversely proportional to the sum of all the kinetic processes: rate constant of fluorescence emission (${\text{k}}_{\text{f}}$), rate constant of FRET (${\text{k}}_{\text{FRET}}$), if present, and rate constant of all other non-fluorescent mechanisms (${\text{k}}_{\text{nf}}$), responsible for relaxation of the excited fluorophore.

(19) $${\text{τ}}_{\text{D}}=\frac{1}{{\text{k}}_{\text{f}}+{\text{k}}_{\text{nf}}},{\text{ τ}}_{\text{DA}}=\frac{1}{{\text{k}}_{\text{f}}+{\text{k}}_{\text{FRET}}+{\text{k}}_{\text{nf}}}$$

where τ_D_ and τ_DA_ are the fluorescence lifetime of the donor in the absence and presence of the acceptor (*i.e*., FRET), respectively.

A convenient way to measure fluorescent lifetime is to determine fluorescence emission decay which follows first-order kinetics for a simple fluorophore [101]. Fluorescence lifetime is mathematically defined as the time taken by a population of excited molecules to decay by a factor of *e* (or to 37% of the initial population) [62,102]. Each fluorophore has its own characteristic fluorescence decay pattern like unique spectral fingerprints. FRET introduces a new de-excitation pathway and consequently accelerates the relaxation process. Therefore, with the increase in the rate of FRET, the donor lifetime decreases because of proximity to acceptors [82]. Understandably, fluorescence lifetime provides a direct measure to determine FRET. For an excited fluorophore, the rate of return to the ground state depends on their number in the excited state times a rate constant, therefore, “E” can be expressed from fluorescence lifetimes as below:

(20) $$\frac{\text{d}[{\text{D}}^{*}]}{\text{dt}}=-[{\text{k}}_{\text{f}}+{\text{k}}_{\text{nf}}][{\text{D}}^{*}]=-\frac{1}{{\text{τ}}_{\text{D}}}[{\text{D}}^{*}]$$

(21) $$\frac{\text{d}[{\text{D}}_{\text{FRET}}^{*}]}{\text{dt}}=-[{\text{k}}_{\text{f}}+{\text{k}}_{\text{FRET}}+{\text{k}}_{\text{nf}}][{\text{D}}_{\text{FRET}}^{*}]=-\frac{1}{{\text{τ}}_{\text{DA}}}[{\text{D}}_{\text{FRET}}^{*}]$$

(22) $$\text{E}=\frac{{\text{k}}_{\text{FRET}}}{{\text{k}}_{\text{FRET}}+{\text{k}}_{\text{f}}+{\text{k}}_{\text{nf}}}=\frac{{\text{k}}_{\text{FRET}}+{\text{k}}_{\text{f}}+{\text{k}}_{\text{nf}}-\left({\text{k}}_{\text{f}}+{\text{k}}_{\text{nf}}\right)}{{\text{k}}_{\text{FRET}}+{\text{k}}_{\text{f}}+{\text{k}}_{\text{nf}}}=1-\frac{{\text{τ}}_{\text{DA}}}{{\text{τ}}_{\text{D}}}$$

Equations (20) and (21) correspond to the first-order decay kinetics of donor only and FRET samples. In the above equations, $\left[{\text{D}}^{*}\right]$ and $\left[{\text{D}}_{\text{FRET}}^{*}\right]$ represent concentration of donors at excited state for donor and FRET samples, respectively, at time “t”. The rate constants (${\text{k}}_{\text{f}}$, ${\text{ k}}_{\text{nf }}$ and ${\text{k}}_{\text{FRET}}$) are described in Equation (19).

Fluorescence lifetime based FRET (FL-FRET) can be carried out in a microscope, a spectrofluorometer or a flow cytometer. An important feature of FL-FRET is its ability to predict the fraction of acceptor-bound donor based on the fluorescence decay curve [19,94]. Fluorescence lifetime is also independent of fluorophore concentrations, emission of acceptors and instrumental factors. Therefore, FL-FRET does not suffer from the major issues seen in intensity analysis based FRET methods like spectral spillover, differences in local concentrations of fluorophores, variation in excitation intensity and exposure duration [35,62,101]. This means that the whole FRET experiment is simplified, requiring less experimental controls and normalization procedures. Therefore, it is highly valuable under conditions where the above experimental parameters are hard to control or determine. Fluorescence lifetime is determined by energetically unstable state of a fluorophore; therefore, it is sensitive to perturbations related to temperature, polarity, refractive index of medium and various quenching effects [35,102]. The exploitation of FL-FRET in microscopy also makes it possible to map the spatial and temporal lifetime dynamics of the molecule with increased accuracy. However, FL-FRET requires long acquisition times in microscopy. Additionally, most biologically relevant fluorophores exhibit lifetimes of nanoseconds. Therefore, fluorescence lifetime measurements also demand sophisticated and expensive instrumentation [103].

### 6.3. Fluorescence Anisotropy Based Approach

Fluorophores are randomly oriented in space and time even in the case of fluorescently labeled plasma membrane protein. Imagining that polarized light excites a stationary fluorophore, the consequent fluorescence emission is also polarized in the same plane. Exposure to a polarized light typically leads to excitation of only a fraction of the total population of fluorophores. This is because fluorophores are free to enjoy any random orientations and only those fluorophores are excited whose absorption transition dipole is aligned suitably or nearly parallel to the polarization plane of the excitation source, a process called photoselection. Furthermore, during the excited state, fluorophores can demonstrate reorientation before they lose energy through both radiative and non-radiative mechanisms including FRET. Consequently, the emitted light is depolarized in comparison with the polarized excitation source [11]. Fluorescence anisotropy (r) defines how much fluorescence emission is polarized after polarized excitation. If a fluorophore is illuminated with a vertically polarized light, then, both vertical (*I*_v_) and horizontal (*I*_h_) emissions should be collected. Processes leading to depolarization of emission are then characterized by these two intensities based on anisotropy calculated according to Equation (23).

(23) $$\text{r}=\frac{I_{\text{v}}-I_{\text{h}}}{I_{\text{v}}+2I_{\text{h}}}$$

Anisotropy is sensitive to the size and shape of the molecule, rigidity or fluidity of the molecular environment, rotational motion and molecular association events [13]. Larger fluorophores will have slower mobility whereas smaller sized fluorophores will tumble and rotate faster. Therefore, larger species will have high anisotropy while small species will have low anisotropy values. However, it is not only the rotation of fluorophores that can alter anisotropy. Typically, FRET also alters the anisotropy of the fluorophore (Figure 3) [11,13]. HeteroFRET shortens the donor's lifetime; therefore, the donor has less time to rotate during the excited state lifetime before emitting a photon. Consequently, donor emission is hyperpolarized (relative to the case when no heteroFRET takes place) resulting in an increase of anisotropy. HomoFRET does not affect lifetime but rather leads to the transfer of energy between like molecules. Each homoFRET step depolarizes the excited population since the acceptors excited by homoFRET are not parallel to the donor. Since the donor and the acceptor are spectroscopically identical, *i.e.*, their fluorescence is indiscriminable, the eventual emission is less polarized with a resultant decrease in anisotropy. Importantly, ensemble fluorescence intensity and lifetime of donor are reduced in heteroFRET but remain unchanged in homoFRET. The extent of depolarization of the fluorophore emission in homoFRET depends on the oligomerization state of the proteins. The larger the number of molecules in a cluster, the lower the anisotropy of the overall fluorescence emission is [13,14]. This feature makes homoFRET a useful tool in the quantitative analysis of large protein clusters [14,104]. However, polarization artifacts induced by sample and instrumental factors easily influence this method. Optical lenses with high numerical aperture (>1) are also found to cause significant depolarization of the emission light. The apparent anisotropy decreases with the increase in numerical aperture of the objective lens. Therefore, objectives with lower numerical aperture are more accurate for anisotropy measurements although with loss in resolution and sensitivity [105]. Anisotropy measurements require highly expressed proteins because the emission signals from the fluorophores are significantly reduced due to the polarizer and also as a result of splitting of the emission signals into vertical and horizontal components [62,106]. Additionally, anisotropy is not very sensitive to the FRET efficiency. It is only suitable for providing information on the presence or absence of FRET, but cannot be used to measure small changes in FRET [62,94]. Nonetheless, both microscopic and flow-cytometric applications of anisotropy can be found in the literature [13,104].

## 7. Applications of FRET in Membrane Biology

The exponential growth in studies applying FRET is explicitly tied to the acceptance of the technique by the biologists. FRET has influenced and impacted different domains of science whether it is molecular biology, cell biology or genetics. It is impossible to document each of these developments here; therefore, we are going to focus on the studies related to membrane biology which has been the subject of our investigation for three decades now. Lectin receptors were the first molecules to be studied *in situ* in the plasma membrane of a cell using FRET in 1976 [107], a couple of years after the proposition of the Singer-Nicholson fluid mosaic model, in 1972 [108]. Coincidentally, the discovery of monoclonal hybridoma technology also occurred at the same time, in 1975 [109]; however, the first study did not involve antibodies as an affinity reagent. Alongside the general acceptance of the cluster of differentiation (CD) classification of monoclonal antibodies starting in 1982 [110], the use of antibodies as probes for FRET has become dominant. The incentive for us to carry out research on cell surface receptors was the conviction that the plasma membrane was the interface for the cell to communicate with its extracellular environment and thus was subjected to dynamic changes, yet its molecules were not randomly distributed, but constantly reorganized as function required. Initial studies were also greatly helped by the availability of monoclonal antibodies against surface receptors of leukocytes. Subsequently, we were able to show that distribution of proteins on the cell surface was non-random and dynamically changing [111]. Some of these findings on membrane molecules of lymphocytes and cancer cells will be re-iterated below to show the utility of FRET in biological systems, and to highlight the related or potential biological functions.

### 7.1. Organization of Antigen Presenting Molecules in the Plasma Membrane of B Cells

Conventionally, it was believed that only peptides were antigenic. However, the perception has changed over the last two decades with a broad range of smaller molecules being found capable of activating T cells. With the antigens also came the diversity in antigen presenting molecules: Major histocompatibility complex I and II (MHC I and MHC II) present peptides, Cluster of Differentiation 1 (CD1) a, b, c, and d present lipid-based antigens, MHC related protein 1 (MR1) presents vitamin metabolites and butyrophilin presents phosphorylated antigen to T cells [112]. In the early 1980s, only peptide antigen presentation was assumed to induce adaptive immune response. Therefore, our initial research was focused on MHC proteins in order to reveal whether they exhibited specific topological features in the plasma membrane of cells. Previous studies demonstrated that antibodies against MHC I would co-cap MHC II molecules on the surface of B lymphocytes. It was an indirect indication of the proximity of MHC I and MHC II molecules [10,113]. Therefore, we decided to apply the more direct approach of FRET to investigate the association between MHC I and MHC II in B lymphoid cells [114,115]. For this purpose, a panel of monoclonal antibodies, conjugated with either FITC (donor) or TRITC (acceptor), and specific for MHC molecules were used. Cells were labeled simultaneously with fluoresceinated and rhodaminated antibodies, then, measurements were performed in a flow cytometer and FRET efficiency was calculated on a cell-by-cell basis (please refer to the section of Three-Channel Emission Measurement). Mean values of FRET efficiency distribution histograms for measured cells were used to ascertain the proximity relationship of MHC molecules. Results suggested that MHC I and MHC II proteins were physically associated already before co-capping. In addition, our FCET studies also revealed that MHC I and MHC II proteins can form homoclusters in the plasma membrane of resting cells [114,115]. Interestingly, MHC II isotypes, HLA-DP, HLA-DQ and HLA-DR, favored inter-isotype association instead of intra-isotype association [114], although intra-isotype association can also be found in B cell lines [114,116]. In a similar FCET approach, but using Alexa 546- and Alexa 647-conjugated antibodies as donors and acceptors, respectively—which is a better FRET-pair than FITC and TRITC—we also documented recently that CD1d, a lipid antigen-presenting molecule, is a part of the membrane domains that contain MHC I and MHC II. MHC I heavy chain and CD1d heavy chain is very similar in structure. Generally, both of these proteins require β_2_-microglobulin (β_2_m) to be bound non-covalently to acquire a functional state. FRET studies and quantitative determination of MHC I, CD1d and β_2_m protein numbers indicated that unlike MHC I, most of the CD1d proteins were free from β_2_m in the plasma membrane of C1R-CD1d cells, a B lymphoid cell line stably expressing CD1d [116]. The functional manifestation of β_2_m free CD1d is obscure, however, β_2_m free CD1d heavy chain has been found to activate T cells in mice [117]. We also noted a decrease in MHC II and increase in MHC I and β_2_m expression on the surface as a result of CD1d expression in this cell line [116]. It should be emphasized that the molecular association of MHC and CD1d proteins in the plasma membrane can influence their biological functions. In this regard, MHC I association with CD1d on the cell surface has been found to diminish the ability of CD1d expressing antigen presenting cells to activate CD1d specific T cells [118] and MHC II surface expression has been observed to enhance CD1d-mediated antigen presentation [119] in previous studies. Further using FCET approach, we have shown that tetraspanin proteins (CD53, CD81 and CD82) were in close proximity of MHC I and MHC II in B cells [115], and subsequently other studies documented the important roles of tetraspanin in antigen presentation [120]. Immunoprecipitation experiments have also revealed the association of CD9 and CD82 with CD1a and CD1d in immature dendritic cells [121] and B cells [122]. Therefore, interaction with tetraspanin molecules seems to be a general feature of antigen presenting molecules. However, the roles of these tetraspanin proteins might differ between the antigen presenting cells owing to their differential impact on surface expression of antigen presenting molecules. For instance, using siRNA mediated gene silencing approach Hoorn *et al*. [123], demonstrated that silencing of CD9, CD63 or CD81 increased MHC II surface expression whereas no effect was observed when CD82 was silenced. The mechanisms responsible for such effects are unclear, nonetheless, it was presumed that the binding of CD63 possibly would delay the release of MHC II from the multivesicular bodies within the cell [123]. Tetraspanin proteins seem to organize into tetraspanin enriched domains in the plasma membrane mediating and recruiting several surface molecules including antigen presenting molecules, leukocyte receptors, integrins and signaling proteins. These proteins thereby are found to influence cellular functions such as intracellular signaling, antigen presentation, and migratory events [120]. Using FCET, we found that MHC I, CD1d and MHC II had different propensities for GM_1_ ganglioside-rich regions, also called lipid rafts. MHC II (or CD1d) showed high FRET efficiency with GM_1_ gangliosides, whereas, MHC I exhibited only a small FRET efficiency. This finding shows that MHC II and CD1d favor the proximity of GM_1_ gangliosides whereas MHC I is primarily located in non-GM_1_ regions [116]. Rafts and tetraspanin domains display several similar attributes; however, differences also seem to exist between them but remain ambiguous. In this regard, FRET studies aiming to define the dynamical organization of these domains relative to MHC I, MHC II and CD1 species before and during antigen presentation can provide valuable information.

### 7.2. Cytokine Receptors and MHC Proteins in T Cells

Application of flow-cytometric FRET to T cell surface also revealed the aforementioned interactions between MHC I and MHC II proteins [124,125] suggesting association of the two groups of MHC as a general feature in the plasma membrane of cells. Interestingly, co-immunoprecipitation studies had previously revealed molecular complexes of CD1a with CD1b, CD1c or MHC I heavy chain in normal thymus cells [126,127]. Based on these findings, it can be postulated that all CD1 isoforms, if expressed, can partially co-exist in similar regions of the plasma membrane inhabited by MHC proteins although it seems that cells may rearrange protein organization under pathological conditions [128]. T cells express diverse set of interleukin receptors responsible for their life and death [129]. Therefore, we extended our FCET studies to demonstrate the proximity of interleukin receptors, IL2R and IL15R, with each other and with MHC proteins in the plasma membrane of T cells [124,130]. IL2 and IL15 receptors comprise three distinct subunits: a unique and cytokine-specific α-chain and the β and γ chains that are shared by both IL2R and IL15R. Due to common β and γ chains, IL2R and IL15R can induce similar biological functions; however, they can also initiate distinct signaling mechanisms. The hetero-trimeric complex of α, β and γ subunits is the high-affinity state for these receptors to bind to their respective ligands IL2 and IL15 [129]. The much debated topic was the assembly of α, β and γ subunits for IL2R and IL15R in T cells. In the case of IL2R, it was assumed that α and β subunits existed separately in the absence of ligand (IL2) and pairing occurred only after the binding of α subunit with IL2. To determine the mechanisms of the assembly of hetero-trimeric complex of IL2R and IL15R, we applied FCET to map the proximity of α, β and γ subunits to each other on Kit 225 K6 human T-lymphoma cells using FITC- and Cy3-conjugated monoclonal antibodies. Experiments on these cytokine receptors revealed positive FRET efficiency between all the three subunits of IL2 and IL15 receptors even in the absence of ligand. These results suggested that at least a fraction of these receptors could exist in a hetero-trimeric high-affinity state in resting human T-lymphoma cells. The addition of cytokines (IL2 and IL15) led to a change in FRET efficiency reflecting the change in the association of receptor subunits. In general, cytokines (IL2 and IL15) led to a tightening of the hetero-trimeric complex formed by the respective receptors. Homo-association and hetero-association was also seen for IL2Rα and IL15Rα both in the absence and presence of IL2 or IL15. Therefore, we proposed a hetero-tetrameric model of IL2/IL15 receptor complex comprising IL2Rα, IL15Rα, and the common β and γ chains. The surrounding environment thus dictates the molecular assembly for high-affinity receptor complex formation for IL2R or IL15R where specific cytokines favor respective high-affinity receptor trimeric complex while the unused α-chain (either IL2Rα or IL15Rα) is nudged from the site of cytokine–receptor interaction [130,131]. Considering the role of interleukin receptors in T cell homeostasis, the molecular organization of IL2R and IL15R in the plasma membrane might be of therapeutic relevance. IL2 and IL5 receptors were found to be upregulated in Crohn’s disease. The increased self-association of IL2Rα receptors but their decreased association with γ chains was revealed by FCET [132]. This indirectly suggests that both IL2Rα and IL15Rα receptor-based trans-presentation—a mechanism in which a surface interleukin receptor (α chain) can present the bound cytokine to nearby cells during cell–cell interaction [129]—could be the dominating cellular function in Crohn’s disease. Evidence of an excessive trans-presentation by IL15Rα during Wegener’s disease, an inflammatory disease similar to Crohn’s disease, supports the above notion [133]. Likewise, association of IL5Rα (or IL2Rα) with MHC I was consistently observed with FCET studies and this molecular association increased during Crohn’s disease. This is additional evidence regarding the potential secondary cellular function of MHC I beside antigen presentation. However, further studies will be required to prove such functions of MHC I.

### 7.3. Dynamic Reorganization of Membrane Proteins in T Cells during Immune Synapse Formation

Antigen recognition by T cells is fundamental in initiating adaptive immune response. For this process, it is necessary that antigen presenting cells (APCs) and T cells come closer to each other and form a functional immunological synapse. The hallmark of synapse formation is the interaction of T cell receptors (TCRs) with the cognate peptide-MHC complex and the simultaneous reorganization of co-stimulatory molecules, adhesion proteins and membranes in both cells [134]. The spatial and temporal details of molecular events occurring at the central region of the synapse have been a subject of significant interest for several years now. FRET has been instrumental in defining these changes for transmembrane molecules and membrane-associated molecules in T cells before and during antigen recognition phase. The earliest demonstration of CD4 and CD3 reorganization in the plasma membrane of CD4 helper T cells was achieved by FCET. The study was not yet based on formation of an immunological synapse between APC and T cells; it was an antibody cross-linking study involving CD3, CD4 and CD45 proteins. Combinations of FITC and TRITC conjugated monoclonal antibodies against CD3, CD4 and CD45 were used as FRET-pairs. Increase in FRET efficiency between CD3/TCR and CD4, but no FRET between CD3/TCR and CD45 was observed when CD4 T cells were activated with anti-CD3 antibodies. This suggests that the immediate response of T cells on antigen recognition possibly would be the redistribution of proteins, with CD3/TCR coming closer to CD4 while CD3/TCR being persistently distant from CD45 [135]. Later, the same group documented that the movement of CD4 towards CD3/TCR was dependent on the interaction of p56^lck^ with the cytoplasmic domain of CD4, especially at positions 420 and 422 [136]. A few years later, Bacso *et al*. [137] demonstrated a possibility of measuring intercellular FRET between APCs and T cells undergoing immune synapse formation making a clever use of donor photobleaching FRET. Exploiting FITC and TRITC combination as a FRET-pair, they showed that in the cytotoxic T cell synapse, FRET occurred between CD8 (T cell) and MHC I (B cell) but not between adhesion molecules LFA1 (T cell) and ICAM1 (B cell). However, they observed a spatial heterogeneity in energy transfer between CD8 and MHC I (0%–30%) at contact regions between conjugates suggesting multiple points with varying degree of interactions. Since they did not observe any FRET between ICAM1 and LFA1, it was presumed that the labels on these adhesion molecules were located farther away than FRET distance (beyond 10 nm) [137].

Today, the synaptic region is defined as a central circle dominated by CD8 or CD4, CD3/TCR, CD28 and LAT proteins (known as central supramolecular activation complex, cSMAC), surrounded by an LFA1 and CD2 rich peripheral supramolecular activation complex (pSMAC), which is further surrounded by the distal SMAC region (dSMAC), containing mostly CD45, CD43 and CD44 molecules [134]. With microscopic FRET based on GFP variants fused to CD3ζ and CD4, Zal *et al*. [52], documented that agonist and antagonist molecules differentially influenced association of TCR/CD3ζ with CD4 in the synaptic region. An increase in FRET efficiency between CD3ζ-CFP and CD4-YFP was observed as a result of MHC II presenting an agonist peptide, whereas, the same was not observed for antagonist peptide despite recruitment of both CD4 and TCR/CD3ζ to the membrane contact regions of APC and T cell. Instead, the antagonist had a negative impact on the close-range interaction between CD3ζ and CD4 formed because of agonist treatment [52]. They later on showed that the recruitment of co-receptor, CD8β-YFP, to the synapse is the result of non-cognate interaction between CD8 and MHC I, as it relied on MHC density, and was independent of antigen unlike TCR movement to the synapse. They observed that non-stimulatory antigen presented simultaneously with an antigenic peptide stimulated association of CD8β-YFP with TCR/CD3-CFP, especially between their cytoplasmic domains during recognition of cognate peptide-MHC proteins [138]. We also found a molecular level interaction between TCR and CD8α using microscopic acceptor photobleaching FRET. In this method, an observation of increased donor fluorescence after photobleaching the acceptors is an indication of molecular proximity. We found that synapses between the cells were not formed in the absence of CD8, corroborating the significance of CD8 in the stabilization of TCR-MHC I interactions. Further, the interaction between TCR and CD3, and CD8 and CD3 was clearly observed but no FRET resulted between TCR and CD45, TCR and CD28, and, CD3 and CD45 within the synaptic region, the observation that is in line with the aforementioned study [139].

We also studied the topological features of CD45 isoforms, a protein tyrosine phosphatase, in the plasma membrane of T cells. Eight different isoforms of CD45 are found in T cells, but only five of them are significantly expressed in T cells. With FCET, we were able to characterize the organization of three of these isoforms, CD45R0, CD45RBC, and CD45RABC, in the plasma membrane of T cells. Homoassociation FRET measurement of cells labeled with 50:50 mixtures of Cy3 and Cy5 conjugated Fab’s antibody fragments against CD45 isoforms revealed significant FRET efficiency only for the CD45R0 isoform. Therefore, CD45R0 but not CD45RBC or CD45RABC was found to exist as homodimers on the cell surface. Comparatively, CD45R0 also preferentially formed heterodimers with CD4 and CD8 proteins. Interestingly, this observation paralleled the results that CD4-associated p56^lck^ tyrosine kinase activity and cellular protein were elevated with a concomitant increase in TCR signaling events in CD45R0 sublines in comparison with CD45RBC expressing sublines. Therefore, a postulation was made on this basis regarding the homodimerization of CD45R0 on the cell surface and the consequentially increased pool of active CD4-associated p56^lck^ tyrosine kinase [140].

### 7.4. Elucidating the Membrane Features of ErbB/HER Kinases

Members of epidermal growth factor (EGF) receptor family, *i.e.*, ErbB proteins, belong to transmembrane receptor tyrosine kinases (RTKs) which are implicated in diverse cellular functions, including proliferation, differentiation and migration. The ErbB family consists of four proteins: ErbB1 (HER1 or EGFR, epidermal growth factor receptor), ErbB2 (HER2), ErbB3 (HER3), and ErbB4 (HER4). The aberrant functioning of ErbB kinases is known to cause cancers of the breast, lung, brain, cervix, ovary, colon *etc.* [82,141]. In general, these proteins exist in various combinations in the plasma membrane. Reorganization and formation of kinase active homo- and hetero-oligomers are known to occur between family members upon ligand binding. Several polypeptide growth factors which are overproduced in tumors can bind to these ErbB proteins, except for ErbB2, leading to induction of intracellular signaling via phosphorylation of cytoplasmic tyrosine residues. Surprisingly heteroassociation of ErbB3, which is kinase deficient, with ErbB2, which has no physiological ligands, has been found to constitute the most potent mitogenic pairing among all ErbB combinations [9,141]. Due to the high expression of these proteins, availability of many model cancer cell lines, and the great therapeutic importance of ErbB proteins, these proteins have been among the most widely studied RTKs. In fact, these proteins present yet another convincing example to show that cellular fate is reflected by the distribution patterns of the molecular species in the membrane. Studies on ErbB proteins using various FRET approaches revealed that all ErbB kinases have formed homo- and hetero-assemblies in the plasma membrane [9,13,82,142]. We wanted to understand the degree of clustering of ErbB1 and ErbB2 receptors in cancer cell lines; therefore, we performed anisotropy-based homoFRET measurements in a flow cytometer. We formulated a theoretical model based on the dependence of fluorescence anisotropy on the fraction of monomers and the number of proteins in a single cluster. The theoretical curves from the model exhibited different anisotropy curves (and values) for different degrees of molecular clustering. Fitting this model to the anisotropy data obtained from cells using anti-ErbB1 or anti-ErbB2 antibodies, we found that the majority of ErbB1 was monomeric in nature, whereas ErbB2 distributed as large homoclusters (~100 proteins) in the plasma membrane in the quiescent state. Stimulation with EGF—a ligand for ErbB1, or pertuzumab—an antibody that blocks ErbB2 dimerization, or heregulin—an ErbB3 or ErbB4 specific ligand caused the redistribution of ErbB1 and ErbB2. EGF and heregulin treatment decreased the homoclusters of ErbB2 whereas ErbB1 clusters showed increase in homoclustering upon EGF treatment [104]. We also noticed a negative correlation between ErbB2 homoassociation and ErbB2 tyrosine phosphorylation [104] or local ErbB3 concentration [143]. Therefore, we were interested in learning how the above treatments would affect the heteroassociation between ErbB1 and ErbB2. To gain insights into this feature, we applied a variant of the acceptor photobleaching method that we termed FRET-sensitized acceptor photobleaching (FSAB) technique to quantitate the ratio of ErbB1 and ErbB2 in their heteroclusters. In FSAB, acceptors within FRET distance of donors are excited at a higher rate than free acceptors owed to the additional excitation by FRET if excitation is carried out in the donor absorption range. Consequently, donor-bound acceptors are photobleached preferentially at a higher rate, and as a result, FRET efficiency drops to zero when all the acceptor molecules in FRET distance are bleached. The remaining acceptor signal is from those acceptors that were not in FRET distance from donors. Therefore, FRET-sensitized acceptor bleaching kinetics can be used for estimating the fraction of acceptors in the vicinity of donors. FSAB revealed that only about 10% of ErbB2 is in heteroclusters with ErbB1 in quiescent cells, and with EGF treatment, the amount of ErbB2 associated with ErbB1 is doubled [64]. This observation links the decrease in homoclustering of ErbB2 and underlies the increased formation of heteroassociation between ErbB1 with ErbB2 as a result of EGF treatment [104]. Overall, many of our FRET studies suggest that the large homoclusters of ErbB2 act as a reservoir, which is used by ErbB1 and ErbB3 to form active heteroclusters upon ligand stimulation. We have also found that ErbB1 and ErbB2 associate with cell adhesion molecules, especially with integrin˗β1. Two-sided FRET [144] measurements that can reveal pairwise interactions among three chosen molecules indicated a degree of complementarity between ErbB homoassociation and its association with integrins, and delineated a correlation between this complementarity and resistance to humanized antibody therapy. Furthermore, FRET analysis of frozen sections from clinical glioblastoma samples has revealed a correlation between such heteroassociation and tumor grade and prognosis, pointing to the possible application of FRET in predictive diagnostics [145].

## 8. Conclusions

FRET is definitely attractive because of its ability to report molecular behavior at a resolution far below the optical diffraction limit. Ironically, the requirement of FRET to have spectral overlap between the FRET-pair is also the source of artifacts. In general, higher spectral overlap yielding a better FRET signal necessitates more extensive corrections for non-FRET signals [68]. Therefore, separating FRET from non-FRET signal is a major challenge. Correcting for non-FRET signals can especially be a problem with fluorescent proteins which generally show broad excitation and emission spectra [37]. FL-FRET is insensitive to fluorophore concentration and spectral overlap, and thus, can be a reasonable alternative. In particular, knowing the photophysics of fluorophores or FPs and the caveats of the used FRET methodology helps to properly interpret the results of FRET experiments and to come-up with the correct workarounds. Many FRET studies use ectopic expression of proteins. In such cases, it is advised to match the expression level of the proteins with the physiological expression levels and to evaluate the effect of fusion tags on protein localization and function to avoid any random molecular interactions. Examining the dependence of “E” on acceptor density or donor-acceptor ratio can also help in deciding whether the observed molecules associate randomly or non-randomly [15]. However, one has to keep in mind that not observing FRET does not necessarily indicate the absence of molecular association in all cases. Absence of FRET could be a result of steric hindrance for neighboring molecules or protein domains or the competition between the used FRET-pair antibodies or incomplete labeling [11,15]. The extreme sensitivity of FRET in the sub-10 nm distances is considered the advantage of FRET, however, it is also its drawback owing to the sharp dependence of energy transfer above or below *R*_0_ making long distance measurements, >10 nm, difficult [60]. To remedy this situation, various strategies have been demonstrated recently for carrying out FRET above 10 nm including the use of multiple acceptors [146] or nanomaterials as acceptors [147]. Dependence of FRET on “*ĸ*^2^” and uncertainty in “*ĸ*^2^” further complicates the calculation of FRET. Fluorophores attached via linker to the probe are free to rotate randomly (dynamic regime) and thus can reduce uncertainty of “*ĸ*^2^”, but not completely [15,60]. Especially for FPs, “*ĸ*^2^” of 2/3 may not hold true because FPs have longer rotational correlation-time, and therefore, are virtually static (static regime) in comparison with organic fluorophores during an excited state. It was shown that assuming a dynamic regime for FP rotation would overestimate the separation between FP FRET-pair significantly (by 10% near 0.5 and by 30% near 0.75 energy transfer efficiencies) when compared with the static regime for FP [148]. Hence, FRET is good at relative but not absolute distance measurements [60]. Additionally, the initial formalism of FRET (Equation (1)) was developed for a fixed system with one-donor and one-acceptor; therefore, it cannot predict “E” accurately in a biological case where several donors and acceptors interact simultaneously. In fact, it has been shown that FRET efficiency would increase when multiple acceptors are available for each donor by increasing the probability of each donor to transfer energy to any of the nearby acceptors [149,150]. One should also be careful while drawing conclusions from FRET efficiency because it is not always translatable to distance, particularly in the case of heterogeneous multi-protein systems. In general, most studies use FRET methods that provide FRET efficiency values as a result of ensemble measurement either of molecular events occurring in each pixel as in microscopy or per cell in flow cytometry. However, single-molecule FRET (smFRET) that requires monitoring of individual molecules for FRET changes can also be performed. A key advantage of smFRET is the possibility to avoid ensemble averaging in samples enabling detection of static heterogeneity, *i.e.*, differences in molecules having various degrees of interactions, or dynamic heterogeneity, *i.e.*, time-dependent changes in molecular associations [151]. Nonetheless, smFRET is not always required and the qualitative ensemble FRET experiments can still serve a wide range of life science studies. Assuming that all the factors except distance can be controlled via experimental conditions, then qualitative information can easily be obtained from the measured apparent FRET efficiency. With the growing list of extensively characterized fluorophores and development of easy to use analytical tools for FRET, we believe that any user with sufficient knowledge in the operation of microscopes or flow cytometer should be able to facilely measure FRET. Overall, FRET can be rewarding if known and applied correctly; however, it can also be a fretting experience if the underlying pitfalls and principles of FRET methods are not well understood leading to all sorts of confusion.
